# Low tidal volume ventilation ameliorates left ventricular dysfunction in mechanically ventilated rats following LPS-induced lung injury

**DOI:** 10.1186/s12871-015-0123-8

**Published:** 2015-10-07

**Authors:** Thomas GV Cherpanath, Lonneke Smeding, Alexander Hirsch, Wim K. Lagrand, Marcus J. Schultz, AB Johan Groeneveld

**Affiliations:** 1 Department of Intensive Care Medicine, Academic Medical Center, University of Amsterdam, Meibergdreef 9, 1105 AZ Amsterdam, The Netherlands; 2 Institute for Cardiovascular Research, VU University Medical Center, Amsterdam, de Boelelaan 1117, 1081 HV Amsterdam, The Netherlands; 3 Department of Cardiology, Academic Medical Center, University of Amsterdam, Meibergdreef 9, 1105 AZ Amsterdam, The Netherlands; 4 Laboratory of Experimental Intensive Care and Anesthesiology (LEICA), Academic Medical Center, University of Amsterdam, Meibergdreef 9, 1105 AZ Amsterdam, The Netherlands; 5 Department of Intensive Care Medicine, Erasmus Medical Center, ‘s-Gravendijkwal 230, 3015 CE Rotterdam, The Netherlands

**Keywords:** Left ventricle, Mechanical ventilation, Tidal volume, Contractility, Lipopolysaccharide, Lung injury

## Abstract

**Background:**

High tidal volume ventilation has shown to cause ventilator-induced lung injury (VILI), possibly contributing to concomitant extrapulmonary organ dysfunction. The present study examined whether left ventricular (LV) function is dependent on tidal volume size and whether this effect is augmented during lipopolysaccharide(LPS)-induced lung injury.

**Methods:**

Twenty male Wistar rats were sedated, paralyzed and then randomized in four groups receiving mechanical ventilation with tidal volumes of 6 ml/kg or 19 ml/kg with or without intrapulmonary administration of LPS. A conductance catheter was placed in the left ventricle to generate pressure-volume loops, which were also obtained within a few seconds of vena cava occlusion to obtain relatively load-independent LV systolic and diastolic function parameters. The end-systolic elastance / effective arterial elastance (Ees/Ea) ratio was used as the primary parameter of LV systolic function with the end-diastolic elastance (Eed) as primary LV diastolic function.

**Results:**

Ees/Ea decreased over time in rats receiving LPS (*p* = 0.045) and high tidal volume ventilation (*p* = 0.007), with a lower Ees/Ea in the rats with high tidal volume ventilation plus LPS compared to the other groups (*p* < 0.001). Eed increased over time in all groups except for the rats receiving low tidal volume ventilation without LPS (*p* = 0.223). A significant interaction (*p* < 0.001) was found between tidal ventilation and LPS for Ees/Ea and Eed, and all rats receiving high tidal volume ventilation plus LPS died before the end of the experiment.

**Conclusions:**

Low tidal volume ventilation ameliorated LV systolic and diastolic dysfunction while preventing death following LPS-induced lung injury in mechanically ventilated rats. Our data advocates the use of low tidal volumes, not only to avoid VILI, but to avert ventilator-induced myocardial dysfunction as well.

## Background

Pneumosepsis resulting in acute lung injury frequently requires mechanical ventilation to ensure adequate gas exchange. However, mechanical ventilation itself can instigate ventilator-induced lung injury (VILI) and aggravate respiratory failure through excessive lung distention at peak inspiration, and repetitive opening and closure of lung units. The use of high tidal volume ventilation has shown to be an important contributing factor of VILI with increased morbidity and mortality [1].

The effect of changes in tidal volume size on stroke volume are in part dependent on right ventricular (RV) preload, contractility and afterload which in turn are affected by lung volume, lung compliance and chest wall compliance among others [2]. These complex interactions make an accurate prediction of the change in RV stroke volume upon increased tidal volume size challenging. Moreover, possible deleterious effects of high tidal volumes on left ventricular (LV) function are largely unknown, partly because of the difficulty of measuring myocardial function independent of changes in loading conditions [3]. Commonly employed indices of LV systolic and diastolic function, the peak pressure change in time during isovolumetric contraction (dP/dt_max_) and isovolumetric relaxation (dP/dt_min_) respectively, are both affected by loading conditions. Alternatively, intraventricular derived pressure-volume loops using a conductance catheter can assess LV contractility and compliance relatively load-independent by constructing end-systolic and end-diastolic pressure-volume relationships [4–6]. Furthermore, LV afterload can be assessed and used for the determination of the relationship between LV contractility and afterload which has been used in various experimental and clinical conditions as the primary parameter for LV function [7–11].

Experimental studies have suggested that high tidal volume ventilation may induce various inflammatory mediators that leak into the circulation causing injury to distant organs including the heart [12, 13], with this effect being amplified in the setting of acute lung injury [14–16]. We therefore hypothesized that low tidal volume ventilation may ameliorate a decline in LV function measured in vivo in a model of lipopolysaccharide (LPS)-induced lung injury.

## Methods

### Animal experiments

The study protocol was approved by the Institutional Animal Care and Use Committee (VU University Medical Center, Amsterdam, the Netherlands) and applied with the Guide for Care and Use for laboratory animals of the National Institutes of Health. Twenty Wistar rats, all males, were intraperitoneal anaesthetized with 85 μg/g ketamine (Alfasan, Woerden, the Netherlands) and 12.5 μg/g midazolam (Pharmachemie BV, Haarlem, the Netherlands), followed by continuous intravenous sedation with 20 μg/g/h ketamine and 1.2 μg/g/h midazolam. The animals were paralyzed with continuous administration of 0.6 μg/g/h pancuronium (Organon, Oss, the Netherlands) to facilitate controlled mechanical ventilation (Avea, Care Fusion, Houten, the Netherlands) after placement of a 14G tracheostoma. A stable body temperature of 37 °C was maintained using a heating pad.

The left carotid artery was used for arterial blood gas analysis and, after zero-referencing against ambient air pressure, arterial blood pressure was continuously measured from which heart rate was derived. A balloon catheter (12060 2.0 F, Edwards Life Sciences, Santa Ana, CA, USA) was positioned through the right internal jugular vein in the inferior vena cava to allow occlusion by means of balloon inflation. A Millar pressure-volume conductance catheter system (1.4 F, Millar instruments, Houston, TX, USA) was inserted through the right carotid artery and placed in the left ventricle. This multi-electrode catheter generates an electric field and measures continuously segmental conductance from which LV relative volume units can be determined. With the addition of a micro manometer on the 1.4 F conductance catheter, LV pressure-volume relationships can be generated even in small animals allowing continuous hemodynamic evaluation of LV systolic and diastolic function including contractile and relaxation parameters that are relatively independent of loading conditions [17, 18].

### Study protocol

During at least 10 min animals were allowed to stabilize following preparation after which baseline measurements were obtained. Animals were then randomized for ventilation with tidal volumes of 6 ml/kg or 19 ml/kg and to receive either 2 μg/g saline-dissolved LPS (L2880, LPS from E. coli 055:B5, Sigma-Aldrich, Buchs, Switzerland) intrapulmonary through a miniature nebulizer (Penn-Century, Wyndmoor, PA, USA) or no LPS. Subsequently, four groups (low tidal volumes (LTV) without LPS; LTV with LPS; high tidal volumes (HTV) without LPS; HTV with LPS) of each 5 animals were studied, all receiving a fraction of inspired oxygen (FiO2) of 40 % with an inspiration : expiration ratio of 1 : 2. Positive end-expiratory pressure (PEEP) was set at 5 cmH_2_O with a respiratory rate of 45/min in the low tidal group, while PEEP was set at 1 cmH_2_O with a respiratory rate of 20/min in the high tidal group to acquire equal mean airway pressure and minute ventilation respectively. The respiratory rate and FiO_2_ were only adjusted if necessary to maintain normocapnia or prevent hypoxia respectively. All other ventilatory settings and drug doses remained unaltered during the 4 h entailing experimental protocol. At the end of the experiment, the animals were sacrificed and myocardial function was measured in an ex vivo study [14].

Heart rate, arterial blood pressure, body temperature and ventilatory pressures, including mean and plateau pressure, were continuously measured. Similarly, LV systolic and diastolic parameters were continuously derived from the pressure-volume conductance catheter. Effective arterial elastance (Ea, mmHg/relative volume units) was defined as end-systolic pressure divided by stroke volume and represents LV afterload with a strong correlation with the gold standard aortic input impedance [7, 19]. The maximum rate of pressure development (dP/dt_max_, mmHg/s) was determined during isovolumetric contraction while the maximum rate of pressure decline (dP/dt_min_, mmHg/s) was measured during isovolumetric relaxation. Averages of the aforementioned LV systolic and diastolic parameters were recorded during steady state and calculated during 3 s covering approximately 15 heart cycles. Arterial blood gas analysis was performed every hour and withdrawn blood was replaced by equal volumes of 0.9 % saline. At every hour the balloon of the catheter positioned in the vena cava was inflated for a maximum of 30 s to diminish venous return and subsequently cardiac preload to enable the construction of the LV end-systolic pressure-volume relationship representing LV end-systolic elastance (Ees). Ees was obtained within a few seconds of vena cava occlusion to prevent sympathetic reflexes that increase ventricular inotropy. This way, Ees describes the maximum pressure that can be developed by the ventricle at any given volume and has shown to be a sensitive index of contractility relatively independent of loading conditions in contrast to dP/dt_max_ [20]. As primary parameter of LV systolic function, the Ees/Ea ratio was derived resembling the relationship between LV contractility and afterload [21]. Finally, the end-diastolic elastance (Eed) was calculated using the LV end-diastolic pressure-volume relationship during vena cava occlusion representing LV compliance as primary parameter of LV diastolic function.

### Statistical analysis

We considered 5 animals per group, with 5 measurements in each animal, as a sufficient number for this physiologic study taken into account that no prior study investigated LV hemodynamics with a pressure-volume conductance catheter in the setting of changes in tidal volume combined with LPS administration. The respiratory, hemodynamic and LV parameters were continuously acquired with PowerLab 16/30 (ADInstruments Ltd, Oxford, UK) displayed using Chart version 5.5.6 (ADInstruments Ltd, Oxford, UK) and analyzed offline with PVAN 3.6 (Millar instruments, Houston, TX, USA). The comparison of baseline measurements between the 4 groups was performed using one-way analysis of variance (ANOVA). The pressure-volume loops during vena cava occlusion were obtained hourly during the 4 h entailing experimental protocol. Responses between the 4 groups were compared by general estimated equations (GEE) with tidal volume ventilation and LPS as factors and time as within-subject variable. To analyze an interaction between tidal volume ventilation and LPS on LV parameters, the interaction term “tidal volume ventilation * LPS” was added to the GEE model. Correlations were calculated using Spearman’s rank correlation coefficients. Analyses were performed using SPSS Statistics version 20.0 (IBM Corporation, New York, NY, USA). Data are presented as means ± SD. A two-sided *p*-value < 0.05 was considered statistically significant. Exact *p*-values are given unless *p* < 0.001.

## Results

### Baseline characteristics

The average weight of the twenty rats was 327 ± 12 g with an average tidal volume of 1.95 ± 0.08 ml in the low tidal volume ventilation group vs. 6.27 ± 0.18 ml in the high tidal volume ventilation group (*p* < 0.001). There were no further differences in respiratory, hemodynamic and LV systolic and diastolic parameters at baseline between the 4 groups except for a higher central venous pressure in rats receiving low tidal volume plus LPS (Table 1).

**Table 1:** Baseline measurements in the rats before randomization (*n* = 20)

Variable	LTV	LTV + LPS	HTV	HTV + LPS	*P*-value
Respiration					
pH	7.31 ± 0.02	7.31 ± 0.03	7.30 ± 0.02	7.32 ± 0.05	0.66
PaCO_2_ (torr)	40.3 ± 4.3	42.5 ± 4.1	42.6 ± 3.6	40.7 ± 3.1	0.68
PaO_2_/FiO_2_ (torr)	564 ± 60	538 ± 45	510 ± 31	525 ± 49	0.36
Pmean (cmH_2_O)	7.6 ± 0.5	7.8 ± 0.4	7.4 ± 0.5	7.8 ± 0.8	0.70
Hemodynamics					
MAP (mmHg)	100 ± 12	122 ± 23	105 ± 16	115 ± 23	0.28
CVP (mmHg)	1.4 ± 0.8	2.9 ± 0.9^a^	1.5 ± 1.2	1.3 ± 0.4	0.045
HR (beats/min)	343 ± 35	407 ± 46	360 ± 26	378 ± 49	0.12
LV parameters					
ESP (mmHg)	151 ± 16	157 ± 16	189 ± 36	174 ± 30	0.13
EDP (mmHg)	21 ± 9	16 ± 13	21 ± 5	18 ± 8	0.78
dP/dt_max_ (mmHg/s)	8043 ± 1689	8928 ± 2729	9912 ± 2709	8809 ± 2150	0.63
dP/dt_min_ (mmHg/s)	−10080 ± 1609	−11917 ± 2954	−9741 ± 2230	−11716 ± 2221	0.35
Ees/Ea	1.74 ± 0.64	2.03 ± 0.56	1.88 ± 0.89	1.74 ± 0.80	0.91

*LTV* low tidal volume ventilation, *LPS* lipopolysaccharide, *HTV* high tidal volume ventilation, *CVP* central venous pressure, *dP/dt*_*max*_ maximum rate of pressure development, *dP/dt*_*min*_ maximum rate of pressure decline, *EDP* end-diastolic pressure, *ESP* end-systolic pressure, *Ees/Ea* end-systolic elastance/effective arterial elastance ratio, *HR* heart rate, *MAP* mean arterial pressure, *PaCO*_*2*_ arterial partial pressure of carbon dioxide, *PaO*_*2*_*/FiO*_*2*_ arterial partial pressure of oxygen/fraction of inspired oxygen ratio, *Pmean* mean airway pressure

^a^CVP was higher in the LTV + LPS group compared to the other groups. Data are mean values ± SD

### Respiratory and hemodynamic parameters

The mean pH throughout the 4 h entailing experiment was lower in LPS-treated rats compared to non-LPS treated rats, with a higher PaCO_2_ in rats receiving low tidal volume plus LPS compared to the other groups (Table 2). The mean airway pressure was higher in rats subjected to high tidal volume ventilation, with a lower PaO_2_/FiO_2_ ratio in the rats with high tidal volume ventilation plus LPS compared to the other groups. The mean arterial pressure and central venous pressure was lower in the rats receiving high tidal volume ventilation, while heart rate was lower in the rats with high tidal volume ventilation without LPS treatment compared to the other groups.

**Table 2:** Respiratory, hemodynamic and left ventricular measurements throughout the experiment in the rats (*n* = 20)

Variable	LTV	LTV + LPS	HTV	HTV + LPS
Respiration				
pH	7.31 ± 0.01	7.26 ± 0.02^a,c^	7.36 ± 0.01^a^	7.28 ± 0.02^a,c^
PaCO_2_ (torr)	39.1 ± 1.7	47.0 ± 3.0^a,c,d^	35.8 ± 1.3	37.8 ± 1.8
PaO_2_/FiO_2_ (torr)	534 ± 36	501 ± 23	485 ± 33	324 ± 28^a,b,c^
Pmean (cmH_2_O)	7.6 ± 0.3	7.8 ± 0.2	8.3 ± 0.2^a,b^	8.9 ± 0.2^a,b,c^
Hemodynamics				
MAP (mmHg)	115 ± 8	116 ± 6	104 ± 6^a,b^	98 ± 8^a,b^
CVP (mmHg)	1.9 ± 0.3	2.2 ± 0.6	1.1 ± 0.3^a,b^	0.7 ± 0.3^a,b^
HR (beats/min)	393 ± 13	413 ± 9	362 ± 12^a,b,d^	403 ± 9
LV parameters				
ESP (mmHg)	184 ± 12^b,d^	154 ± 10	167 ± 12	146 ± 14
EDP (mmHg)	20 ± 4	17 ± 5	18 ± 2	17 ± 3
dP/dt_max_ (mmHg/s)	11740 ± 1114	11763 ± 1251	10343 ± 1000	9014 ± 682^a,b^
dP/dt_min_ (mmHg/s)	−13482 ± 1485	−13072 ± 1103	−11267 ± 990	−8555 ± 1005^a,b,c^
Ees/Ea	1.67 ± 0.33	1.45 ± 0.16	1.33 ± 0.13	0.86 ± 0.11^a,b,c^

*LTV* low tidal volume ventilation, *LPS* lipopolysaccharide, *HTV* high tidal volume ventilation, *CVP* central venous pressure, *dP/dt*_*max*_ maximum rate of pressure development, *dP/dt*_*min*_ maximum rate of pressure decline, *EDP* end-diastolic pressure, *ESP* end-systolic pressure, *Ees/Ea* end-systolic elastance/effective arterial elastance ratio, *HR* heart rate, *MAP* mean arterial pressure, *PaCO*_*2*_ arterial partial pressure of carbon dioxide, *PaO*_*2*_*/FiO*_*2*_ arterial partial pressure of oxygen/fraction of inspired oxygen ratio, *Pmean* mean airway pressure

^a^*P* < 0.05 compared to LTV group. ^b^*P* < 0.05 compared to LTV + LPS group. ^c^*P* < 0.05 compared to HTV group. ^d^*P* < 0.05 compared to HTV + LPS group. Data are mean values ± SE

### Left ventricular systolic and diastolic function

**Fig. 1:**
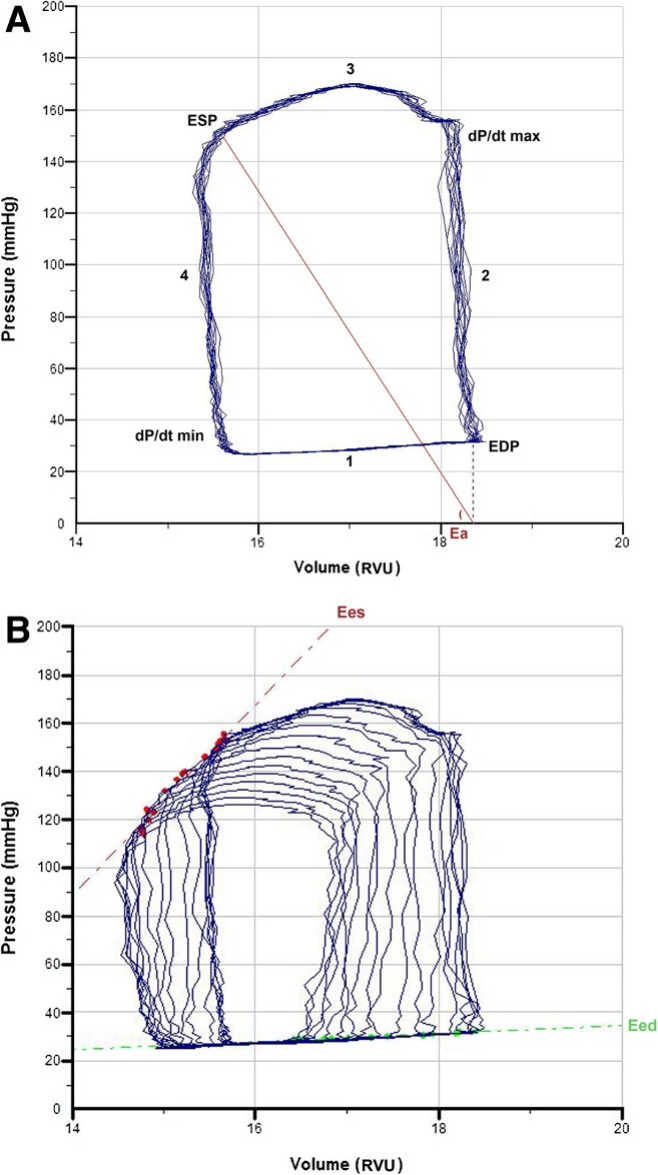
Pressure-volume loops obtained during three seconds showing left ventricular (LV) hemodynamics during steady state (**a**) and vena cava occlusion (**b**). **a** LV function is described during 4 phases of the cardiac cycle: 1) diastolic filling, 2) isovolumetric contraction, 3) ejection and 4) isovolumetric relaxation. EDP = end-diastolic pressure, ESP = end-systolic pressure, Ea = effective arterial elastance which slope is calculated by end-systolic pressure divided by stroke volume, dP/dt_max_ = maximum rate of pressure development during isovolumetric contraction, dP/dt_min_ = maximum rate of pressure decline during isovolumetric relaxation. **b** By decreasing preload through vena cava occlusion, the pressure-volume loops move to the left and become smaller, enabling the measurement of end-systolic elastance (Ees) by the pressure-volume relationship at end-systole. Simultaneously, end-diastolic elastance (Eed) can be measured by the pressure-volume relationship at end-diastole. A representative sample trace as displayed and analyzed by PVAN 3.6 is shown

**Fig. 2:**
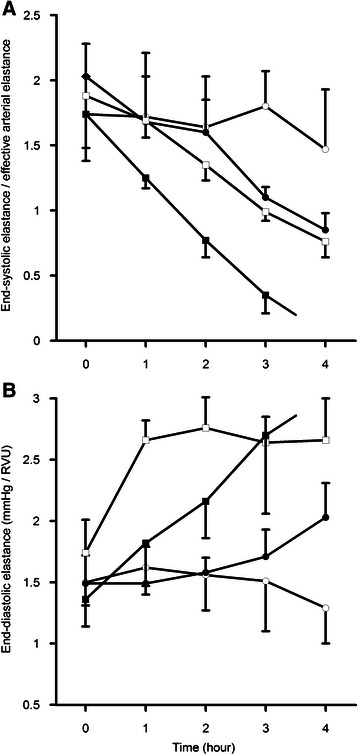
Course of left ventricular end-systolic elastance/effective arterial elastance (Ees/Ea) (A) and end-diastolic elastance (Eed) (B) over time in the 4 rat groups. (○: low tidal volume (LTV), ●: LTV + lipopolysaccharide (LTV + LPS), □: high tidal volume (HTV), ■: HTV + LPS. **a** Ees/Ea decreased over time in all groups (*p* < 0.001) except for the LTV group (*p* = 0.428). A decrease in Ees/Ea was observed in the rats subjected to high tidal volume ventilation vs. low tidal volume ventilation (*p* = 0.007), as well in the rats receiving LPS vs. non-LPS (*p* = 0.045). **b** Eed remained unchanged over time in the LTV group (*p* = 0.223), but increased in the LTV + LPS (*p* = 0.036), HTV (*p* < 0.001) and HTV + LPS (*p* = 0.001) group. An interaction between tidal ventilation and LPS was found for Ees/Ea and Eed (*p* < 0.001) and all the rats receiving HTV + LPS died before the end of the experiment. A *p*-value < 0.05 was considered statistically significant. Error bars represent ± SE

LV systolic and diastolic parameters could successfully be derived from the pressure-volume loops in the rats during steady state (Fig. 1a) and during balloon inflation of the vena cava catheter (Fig. 1b). Ees/Ea decreased over time in rats receiving LPS compared to non-LPS treated rats (*p* = 0.045), as well as in rats subjected to high tidal volume ventilation vs. low tidal volume ventilation (*p* = 0.007) (Fig. 2a). Mean Ees/Ea was lower in the rats with high tidal volume ventilation plus LPS compared to the other groups (*p* < 0.001) (Table 2). Furthermore, mean dP/dt_max_ was lower in the rats with high tidal volume ventilation plus LPS compared to the rats with low tidal volume ventilation. Mean end-systolic pressure was higher in the rats receiving low tidal volume ventilation without LPS compared to LPS-treated rats.

Eed increased over time in the rats receiving LPS with low tidal volume ventilation (*p* = 0.036) and during high tidal volume ventilation (*p* = 0.001) with an increase in the Eed slope representing decreased LV compliance (Fig. 2b). In the rats without LPS administration, Eed remained unchanged over time during low tidal volume ventilation (*p* = 0.223), but increased during high tidal volume ventilation (*p* < 0.001). No differences in mean end-diastolic pressure was observed, yet mean dP/dt_min_ was lower in the rats subjected to high tidal volume ventilation plus LPS compared to the other groups (Table 2). Tidal volume was significantly correlated with Ees/Ea and Eed (*r*^2^ = −0.34; *p* = 0.001 and *r*^2^ = 0.39; *p* < 0.001 respectively), as were the correlations between plateau pressure and Ees/Ea as well as Eed (*r*^2^ = −0.47; *p* < 0.001 and *r*^2^ = 0.42; *p* < 0.001 respectively).

### Survival

A significant interaction (*p* < 0.001) was found between tidal ventilation and LPS for Ees/Ea and Eed. Three rats receiving high tidal volume ventilation plus LPS did not survive beyond 2,5 h, while the 2 remaining rats from that group died at 3.5 h. All rats from the other groups survived till the end of the experiment.

## Discussion

We investigated whether tidal volume size affects LV function in a two-hit animal model of high tidal volumes and lung injury induced by intrapulmonary administered LPS. Our data show that high tidal volume ventilation resulted in a progressive decline in LV systolic and diastolic function over time, especially during LPS-induced lung injury, yet LV function remained unchanged in rats receiving low tidal volume ventilation without LPS. An interaction between tidal volume ventilation and LPS was observed for LV contractile and relaxation parameters implying that the LPS-induced lung injury exerted an additional detrimental effect on LV function possibly contributing to the death of all the rats subjected to high tidal volume ventilation plus LPS administration. Therefore, low tidal volumes may be advocated to prevent VILI as well as to ameliorate LV dysfunction.

Conventional hemodynamic parameters have shown to poorly reflect LV function in sepsis [22, 23]. Even during elevated cardiac output, LV dysfunction is frequently present [24]. Furthermore, cardiac output may not predict mortality in sepsis [25], whereas myocardial depression is thought to characterize the fatal course of septic shock [26, 27]. This paradox may in part be explained by the fact that cardiac output can remain unaltered despite LV systolic dysfunction when a concomitant decreased LV afterload is present [28]. Therefore, LV systolic function should be evaluated in relationship with LV afterload, explaining the common use of Ees/Ea as primary parameter for LV systolic function [7–11]. A mean Ees/Ea of > 1.6 has been associated with a normal LV systolic function while a ratio < 1.0 correlated with a severely impaired LV systolic function [10]. LV systolic function appears to deteriorate at the early phase of septic shock [29]. We observed a progressive decrease in Ees/Ea with a ratio < 1.0 in the rats subjected to high tidal volume ventilation plus LPS, with a lower dP/dt_max_ compared to rats receiving low tidal volume ventilation. Furthermore, end-systolic pressure was lower in LPS-treated rats compared to non-LPS treated rats receiving low tidal volume ventilation.

Besides evidence of systolic dysfunction during sepsis, there are also several reports in the literature demonstrating diastolic dysfunction [30–32]. In our study, LV diastolic function as assessed by Eed was progressively impaired over time in rats subjected to high tidal volume ventilation and/or LPS administration, with the lowest dP/dt_min_ during high tidal volume ventilation plus LPS. Nevertheless, end-diastolic pressure remained unaltered corresponding with a previous described preservation of normal filling pressures during sepsis [28].

Surprisingly, in comparison to the well-known deleterious effects on RV function [33, 34], little is known about the effect of tidal volume size on LV function. Therefore the contribution of LV dysfunction in the observed decrease in cardiac output during high tidal volume ventilation in previous studies remains largely unknown [35, 36]. To our knowledge, the only study specifically studying LV function during changes in tidal volume was performed in pigs by Renner et al. [37]. In this study no decrease in LV function, measured by echocardiography-derived myocardial performance index, was seen during low tidal volume ventilation with 5 ml/kg in contrast to high tidal volume ventilation with 10 ml/kg and 15 ml/kg. In accordance, in our non-LPS treated rats no decrease in relatively load-independent LV systolic and diastolic function was observed during low tidal volume ventilation with 6 ml/kg in contrast to high tidal volume ventilation with 19 ml/kg. The concept that LV function can deteriorate during high tidal ventilation in a load-independent manner is enhanced by the fact that positive end-expiratory pressure, while affecting ventricular loading conditions as well, does not directly alter LV function [38, 39].

A possible explanation for the decrease in LV function upon high tidal ventilation may be the ventilation-induced inflammation that may not only damage the lung, but has shown to injure distant organs as well contributing to morbidity and mortality [40–42]. We used intrapulmonary administration of nebulized LPS which, together with the application of high tidal volume ventilation, created a severe model of acute lung injury with a decrease in PaO_2_/FiO_2_ ratio. The observed aggravation of acidosis in the LPS-treated animals could be explained by the induction of sepsis, which has repeatedly shown to decrease ventricular contractility load-independently assessed by pressure-volume loops [43–45]. Moreover, extrapulmonary organ dysfunction induced by VILI is thought to depress myocardial function rather similar to sepsis [46], such that a cumulative effect on the heart can be expected. We indeed observed an aggravation of myocardial dysfunction induced by high tidal volume ventilation during LPS-induced lung injury potentially contributing to the death of all rats before the end of the experiment. Interestingly, these findings are confirmed ex vivo where high tidal volumes were applied in combination with intrapulmonary LPS [14], in contrast to where LPS was administered intraperitoneal [47], supporting the additional detrimental effect of high tidal volume ventilation in the setting of acute lung injury.

Injurious lung effects of high tidal volume ventilation are increasingly recognized since a large ARDS Network trial showed an absolute reduction in mortality of 10 % when lower tidal volumes were compared with conventional (i.e., high) tidal volumes [1]. Our data show a correlation between tidal volumes and LV systolic and diastolic function suggesting that low tidal volume ventilation may mitigate LV dysfunction beyond its protective effects against VILI. Plateau pressure, another key component of lung protective ventilation, correlated even slightly better with LV function. One could therefore argue that the survival benefit seen in prior lung protective ventilation trials may be explained not only by a reduction in VILI, but in addition by a reduction in concomitant “VIMD”: Ventilator-Induced Myocardial Dysfunction.

Several limitations in our study should be discussed. We looked at LV systolic and diastolic function, but did not measure RV function which could have shed light on the overall effect on cardiac function. Although RV dysfunction induced by high tidal volume ventilation could have contributed to changes in LV loading conditions, this could not fully explain the decrease in LV systolic and diastolic function parameters that are relatively load-independent. It has already been documented that myocardial depression during sepsis affects both ventricles simultaneously [24]. We focused on LV systolic and diastolic parameters as little is known about the effect of high tidal volume ventilation on LV function in comparison to the notorious effects on RV function. Mean airway pressure became higher during the experiment in the rats subjected to high tidal volume ventilation despite application of lower PEEP compared to the low tidal volume ventilation group. Therefore the observed LV systolic and diastolic dysfunction may in part be explained by mechanical ventilator-induced lung injury due to increased airway pressure. Detrimental effects of low PEEP on lung volume as well as an increase in lung injury from greater dynamic strain cannot be excluded [48], although PEEP has predominantly failed to demonstrate a decrease in LV function in the literature [3, 38, 39]. While the rats ventilated with 6 ml/kg showed amelioration of LV dysfunction, the ideal tidal volume size cannot be determined from our data. Left ventricular hemodynamics were measured intraventricular with a pressure-volume conductance catheter as has been previously done in animal models during either changes in tidal volumes or during LPS administration, although not during both interventions [49, 50]. The pressure-volume conductance catheter, when zeroed to atmospheric pressure, provides absolute pressure values in millimeters of mercury, but offers volumes in relative volume units unless external calibration is performed for which the thermodilution technique can be used. Unfortunately, simultaneous thermodilution measurements proved to be infeasible in our rats with already extensive instrumentation. Nevertheless, the ratio of Ees/Ea would not be influenced by the usage of relative volume units while the other systolic (dP/dt_max_ and end-systolic pressure) and diastolic (dP/dt_min_ and end-diastolic pressure) parameters were all pressure derived. However, the Eed value is subjective to the usage of relative volume units, so only the Eed trend over time and not the absolute values could be analyzed. Heart rates and respiratory rates were naturally greater in our small animal model then seen in human, but with a fairly comparable 7:1 heart-lung interaction ratio. Nonetheless, further research in humans need to be undertaken to confirm the findings of our study.

## Conclusions

High tidal volume ventilation decreases LV function, which is aggravated during LPS-induced lung injury in mechanically ventilated rats. Previously, the use of low tidal volumes was advocated to reduce ventilator-induced lung injury. Our data suggests that low tidal volumes preserve LV systolic and diastolic function as well during acute lung injury, ameliorating so-called ventilator-induced myocardial dysfunction.

### Key messages

- In mechanically ventilated rats, both high tidal volume ventilation as well as lung injury induced by intrapulmonary administered LPS decreased LV systolic and diastolic function.
- High tidal volume ventilation exerted an additional detrimental effect on LV function in the LPS-treated rats, none surviving the experiment in contrast to low tidal volume ventilation.
- Low tidal volumes may be advocated to ameliorate ventilator-induced myocardial dysfunction besides ventilator-induced lung injury.

## Abbreviations

VILI: Ventilator-induced lung injury
LV: Left ventricular
RV: Right ventricular
dP/dt_max_: Peak pressure change in time during isovolumetric contraction
dP/dt_min_: Peak pressure change in time during isovolumetric relaxation
LPS: Lipopolysaccharide
LTV: Low tidal volumes
HTV: High tidal volumes
FiO_2_: Fraction of inspired oxygen
PEEP: Positive end-expiratory pressure
Ea: Effective arterial elastance
Ees: End-systolic elastance
Eed: End-diastolic elastance
PaCO_2_: Arterial partial pressure of carbon dioxide
PaO_2_: Arterial partial pressure of oxygen
VIMD: Ventilator-induced myocardial dysfunction
